# Anaerobic Degradation of Syringic Acid by an Adapted Strain of Rhodopseudomonas palustris

**DOI:** 10.1128/AEM.01888-19

**Published:** 2020-01-21

**Authors:** J. Zachary Oshlag, Yanjun Ma, Kaitlin Morse, Brian T. Burger, Rachelle A. Lemke, Steven D. Karlen, Kevin S. Myers, Timothy J. Donohue, Daniel R. Noguera

**Affiliations:** a Great Lakes Bioenergy Research Center, Wisconsin Energy Institute, University of Wisconsin—Madison, Madison Wisconsin, USA; b Department of Civil & Environmental Engineering, University of Wisconsin—Madison, Madison, Wisconsin, USA; c Department of Biochemistry, University of Wisconsin—Madison, Madison, Wisconsin, USA; d Department of Bacteriology, University of Wisconsin—Madison, Madison, Wisconsin, USA; University of Tokyo

**Keywords:** *Rhodopseudomonas*, anaerobic aromatic degradation, aromatic compounds, biodegradation, lignin, photoheterotrophic, syringic acid, vanillic acid

## Abstract

While lignin represents a major fraction of the carbon in plant biomass, biological strategies to convert the components of this heterogeneous polymer into products of industrial and biotechnological value are lacking. Syringic acid (3,5-dimethoxy-4-hydroxybenzoic acid) is a by-product of lignin degradation, appearing in lignocellulosic hydrolysates, deconstructed lignin streams, and other agricultural products. Rhodopseudomonas palustris CGA009 is a known degrader of phenolic compounds under photoheterotrophic conditions via the benzoyl coenzyme A (CoA) degradation (BAD) pathway. However, R. palustris CGA009 is reported to be unable to metabolize *meta*-methoxylated phenolics, such as syringic acid. We isolated a strain of R. palustris (strain SA008.1.07), adapted from CGA009, which can grow on syringic acid under photoheterotrophic conditions, utilizing it as a sole source of organic carbon and reducing power. An SA008.1.07 mutant with an inactive benzoyl-CoA reductase structural gene was able to grow on syringic acid, demonstrating that the metabolism of this aromatic compound is not through the BAD pathway. Comparative gene expression analyses of SA008.1.07 implicated the involvement of products of the *vanARB* operon (*rpa3619*, *rpa3620*, *rpa3621*), which has been described as catalyzing aerobic aromatic ring demethylation in other bacteria, in anaerobic syringic acid degradation. In addition, experiments with a *vanARB* deletion mutant demonstrated the involvement of the *vanARB* operon in anaerobic syringic acid degradation. These observations provide new insights into the anaerobic degradation of *meta*-methoxylated and other aromatics by R. palustris.

**IMPORTANCE** Lignin is the most abundant aromatic polymer on Earth and a resource that could eventually substitute for fossil fuels as a source of aromatic compounds for industrial and biotechnological applications. Engineering microorganisms for the production of aromatic-based biochemicals requires detailed knowledge of the metabolic pathways for the degradation of aromatics that are present in lignin. Our isolation and analysis of a Rhodopseudomonas palustris strain capable of syringic acid degradation reveal a previously unknown metabolic route for aromatic degradation in R. palustris. This study highlights several key features of this pathway and sets the stage for a more complete understanding of the microbial metabolic repertoire required to metabolize aromatic compounds from lignin and other renewable sources.

## Abstract (precis)

Lignin is the most abundant aromatic polymer on Earth and a resource that could eventually substitute for fossil fuels as a source of aromatic compounds for industrial and biotechnological applications. Engineering microorganisms for the production of aromatic-based biochemicals requires detailed knowledge of the metabolic pathways for the degradation of aromatics that are present in lignin. Our isolation and analysis of a Rhodopseudomonas palustris strain capable of syringic acid degradation reveal a previously unknown metabolic route for aromatic degradation in R. palustris. This study highlights several key features of this pathway and sets the stage for a more complete understanding of the microbial metabolic repertoire required to metabolize aromatic compounds from lignin and other renewable sources.

## INTRODUCTION

As one of the major biopolymers present in plant tissues, lignin has the potential to serve as a renewable source of carbon for the biomass-based production of compounds that are currently derived from petroleum. Unfortunately, the ability to derive chemicals of commercial, chemical, or medicinal value from lignin is limited by a lack of the information needed to improve the biological conversion of the aromatics in lignin into valuable products. We are interested in improving our understanding of how bacteria metabolize the aromatic building blocks in lignin and using this information to develop strategies that allow the conversion of this major component of plant cell walls into valuable products.

Syringic acid and other *meta*-methoxy-substituted phenolic compounds are plant-derived aromatics that present both a hindrance and a potential source of value to the chemical, fuel, and biotechnology industries (1–3). Originating from the guaiacyl (coniferyl alcohol) and syringyl (sinapyl alcohol) phenylpropanoids that are polymerized into lignin during secondary cell wall formation (1), *meta*-methoxylated aromatics are frequently present in products generated from deconstructed biomass (4). While present at low concentrations in sugar-rich lignocellulosic hydrolysates, these methoxylated aromatics can nonetheless induce stress responses (2, 5) and cause toxicity (6, 7) in non-aromatic-degrading microbes, leading to decreases in both microbial growth and biofuel yield during fermentation (8, 9). Further, these phenolics are present at much higher concentrations in solubilized lignin streams produced with emerging technologies (10–13). Incorporation of *meta*-methoxylated aromatics into the metabolism of an appropriate, genetically tractable microorganism could provide a promising and efficient route for monolignol valorization through the identification and optimization of the biochemical pathways involved.

To expand the ability of microbes to metabolize syringic acid and related plant-derived aromatic compounds, we are studying Rhodopseudomonas palustris, a metabolically versatile, well-characterized, and genetically tractable purple nonsulfur alphaproteobacterium (14–16) that has a proven and well-understood ability to utilize aromatic monomers (17, 18). Under anaerobic conditions, R. palustris uses the benzoyl coenzyme A (CoA) degradation (BAD) pathway to cleave the aromatic ring of monoaromatic compounds after activation of the molecule via coenzyme A ligation (19). The diversity of aromatic compounds that R. palustris can degrade depends on the existence of accessory pathways that transform aromatic monomers to the common BAD pathway intermediates benzoyl-CoA or 4-hydroxybenzoyl-CoA (20, 21). In addition, previous studies have shown that the growth of R. palustris in lignocellulosic hydrolysates that contain a mixture of plant-derived organic compounds allows for the degradation of aromatic monomers that do not support growth when supplied as the sole carbon source in defined medium (21).

Here we describe studies aimed at understanding the metabolism of syringic acid by an adapted R. palustris strain. By supplying syringic acid to a series of successive cultures, we isolated a strain of R. palustris capable of utilizing this *meta*-methoxylated aromatic as the sole source of organic carbon. We analyzed the degradation of syringic acid by this adapted isolate, R. palustris SA008.1.07, in defined laboratory medium to provide insight into the mechanisms involved in the degradation of this aromatic monomer.

## RESULTS AND DISCUSSION

### Isolation of a syringic acid-degrading R. palustris strain.

**FIG 1:**
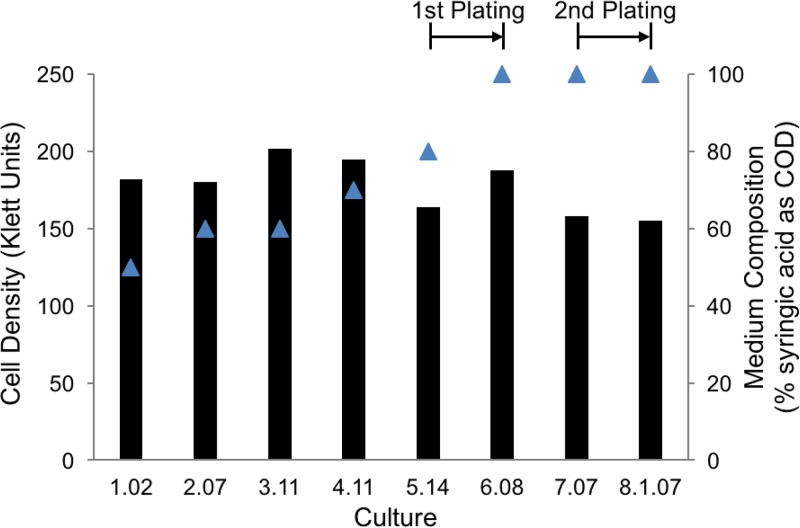
Final cell density (bars; Klett units) and percentage of syringic acid in the culture medium (blue triangles) during sequential anaerobic incubations. Culture 1.02 was started from a colony of R. palustris CGA009 that did not exhibit significant metabolism or growth on syringic acid as a sole carbon source. Each culture was seeded from a subculture of the prior one, except in the two instances indicated as 1st plating and 2nd plating in the figure. Cells were plated and single colonies were selected for isolation prior to the inoculation of cultures 6.08 and 8.1.07. The initial COD of the medium, used as a measurement of bioavailable organic carbon, was maintained at 1 g COD/liter in all cultures by decreasing the proportion of benzoic acid and 4-HBA upon increases in the syringic acid concentration. All cultures were grown anaerobically at 30°C in sealed glass tubes under constant illumination.

**FIG 2:**
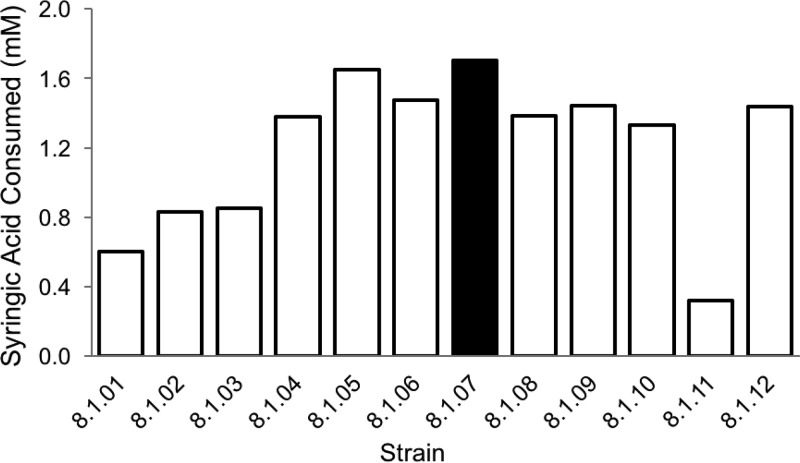
Syringic acid consumption by 12 strains isolated from culture 7.07 (Fig. 1). Strain SA008.1.07 had the highest syringic acid transformation and was selected for further study. The initial concentration of syringic acid in these cultures was 3.47 mM.

R. palustris CGA009 is reported to be unable to grow photoheterotrophically with syringic acid as the sole organic carbon source (14). To explore the potential for R. palustris to evolve the capacity to degrade syringic acid, we established a series of anaerobic cultures in which CGA009 was provided with a combination of syringic acid, benzoic acid, and 4-hydroxybenzoic acid (4-HBA), with the last two being established growth substrates for this strain (22, 23). Cultures were kept under illumination and anaerobic conditions for at least 1 week after growth had reached stationary phase. At the conclusion of each growth phase, extracellular samples from each culture were assayed for the presence of aromatic acids. Cultures showing some decrease in the extracellular syringic acid concentration were used as an inoculum for new cultures containing an equal or higher proportion of syringic acid in the medium (Fig. 1). This process was iterated five times with increases in the proportion of syringic acid in the medium until cells were growing on medium in which syringic acid represented 80% of the organic carbon added (measured as chemical oxygen demand [COD]). The highest-performing culture at this stage, as determined by total syringic acid consumption from the medium (culture 5.14 in Fig. 1), was plated photoheterotrophically onto solid medium containing this compound as the sole source of organic carbon, and 14 colonies were picked after 2 weeks of incubation. The isolated colonies were then used to inoculate separate liquid photoheterotrophic cultures containing syringic acid as the sole source of organic carbon, and the highest performing of these cultures were incubated in a second round of liquid photoheterotrophic growth on medium containing syringic acid as the sole organic carbon source. From a second anaerobic plating (from culture 7.07 in Fig. 1), 12 colonies were obtained. To further test that these cells acquired the ability to grow solely on syringic acid, cells in isolated colonies were first grown photoheterotrophically on succinate and then subcultured to a medium containing syringic acid as the sole photoheterotrophic carbon source. The isolate that degraded the most syringic acid under photoheterotrophic conditions (Fig. 2), hereafter referred to as strain SA008.1.07, was selected for further testing.

### Identification of DMBQ as a compound that accumulates extracellularly during growth on syringic acid by SA008.1.07.

**FIG 3:**
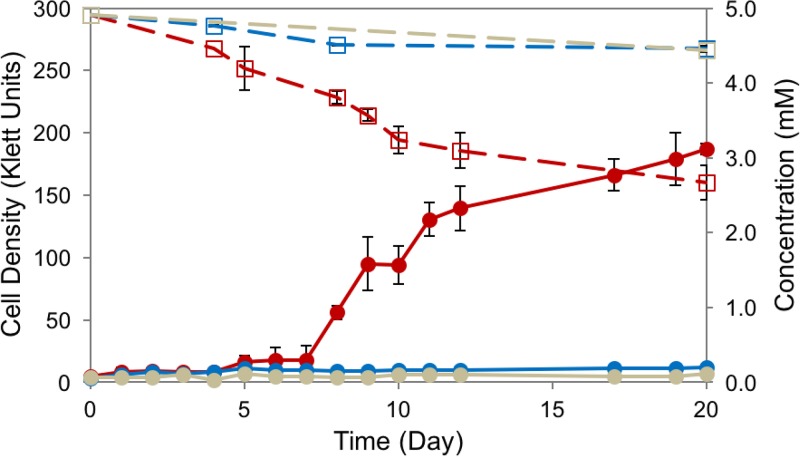
Anaerobic growth of R. palustris SA008.1.07 (red) on 5 mM syringic acid compared to that of parent strain CGA009 (blue) and a light-exposed abiotic control (gray). Solid lines show growth (in Klett units) (●), and dashed lines track the concentrations of syringic acid (□). SA008.1.07 consumed approximately half of the syringic acid initially present in the medium, while CGA009 did not grow on syringic acid. Error bars represent standard deviations from experiments performed in triplicate.

We found that when SA008.1.07 used syringic acid as a sole source of organic carbon under anaerobic, photoheterotrophic conditions (Fig. 3), an orange-yellow tint appeared during early stages of culture growth. However, as growth progressed, the color of the culture became dark and distinguishable from the deep-red color of the accumulating biomass.

**FIG 4:**
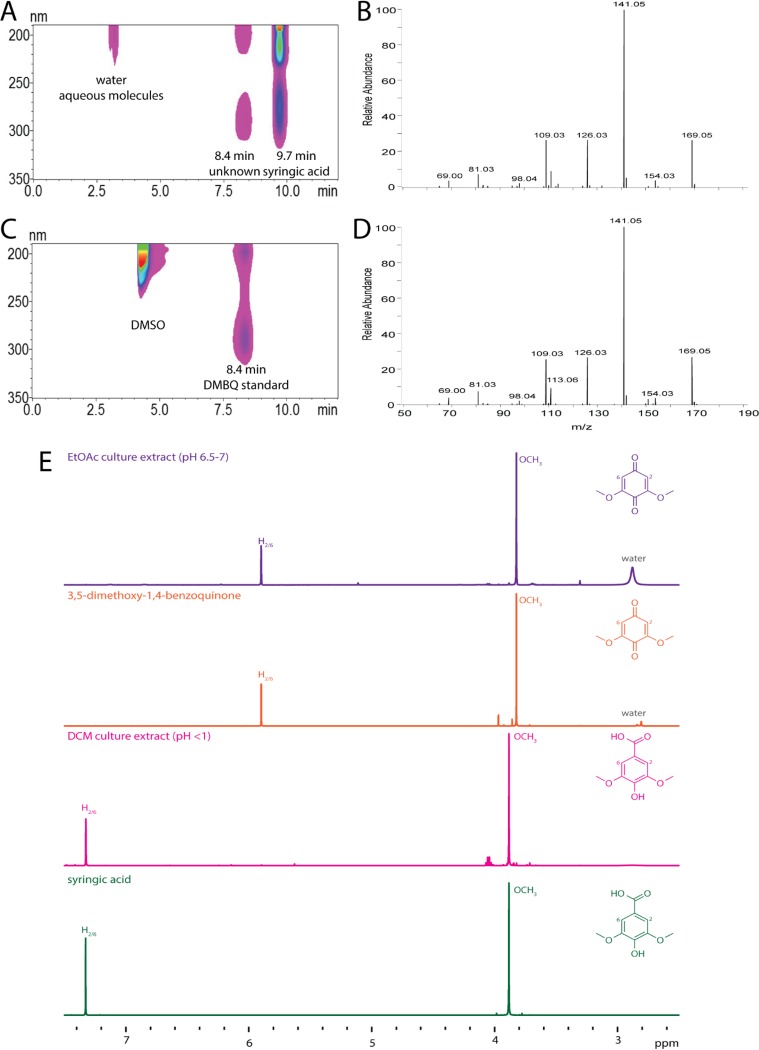
Identification of DMBQ as a soluble extracellular product of SA008.1.07 grown on syringic acid cultures. (A) HPLC contour view of PM-syringic acid medium after SA008.1.07 growth, showing peaks at 8.4 and 9.7 min, with the latter peak corresponding to syringic acid. (B) An LC-MS/MS trace of the compound isolated from the peak collected at 8.4 min suggests an *m/z* ratio of 169.04 g/mol (molecular weight, ∼168 g/mol). (C) HPLC contour view of the DMBQ standard, showing that the retention time matches that of the unknown peak in panel A. The peak at 4 min is DMSO. (D) LC-MS/MS trace of commercially purchased DMBQ, showing a match to the MS spectrum of the unknown peak in panel B. (E) NMR trace of EtOAc-extracted culture medium, an authentic DMBQ standard, DCM-extracted culture medium, and an authentic syringic acid standard.

High-performance liquid chromatography (HPLC) analysis of the medium before and after the growth of SA008.1.07 revealed the accumulation of a light-absorbing unknown product that eluted at 8.4 min (Fig. 4A). By analyzing standards of aromatics that are known or potential syringic acid degradation by-products (3-*O*-methyl gallic acid, gallic acid, vanillic acid, protocatechuic acid) by HPLC, we determined that none of these compounds were found at detectable levels in supernatants from SA008.1.07 cultures. A liquid chromatography-tandem mass spectrometry (LC-MS/MS) examination of the extracellular unknown indicated an *m/z* ratio of 169.05 (Fig. 4B). For further analysis of this unknown, an extractive procedure was performed on the medium, partitioning the compounds into ethyl acetate (EtOAc) or dichloromethane (DCM) (see Materials and Methods), and both fractions were analyzed by nuclear magnetic resonance (NMR). Syringic acid was identified as the major product in the ^1^H NMR of the DCM extract, based both on its spectrum and on a comparison to that of a commercially purchased standard (Fig. 4E). The ^1^H NMR of the EtOAc extract (Fig. 4E) contained two major peaks, indicative of methoxy groups and hydrogen atoms on an aromatic ring. Neither of these signals were split, indicating a lack of coupling to adjacent hydrogen atoms in the compound. The predicted molecular weight of the unknown (∼168.05 g/mol, based on the positive ionization mass spectrometry [MS] spectrum) and the ^1^H NMR pattern suggested that 3,5-dimethoxy-1,4-benzoquinone (DMBQ) was the compound that accumulated during growth on syringic acid. Indeed, NMR (Fig. 4E) and MS analysis (Fig. 4D) of a commercial DMBQ standard, which also has an orange-yellow tint (CAS number 530-55-2), showed that it was indistinguishable from the extracellular product that accumulates when SA008.1.07 is grown on syringic acid.

### DMBQ inhibits the growth of R. palustris SA008.1.07.

**FIG 5:**
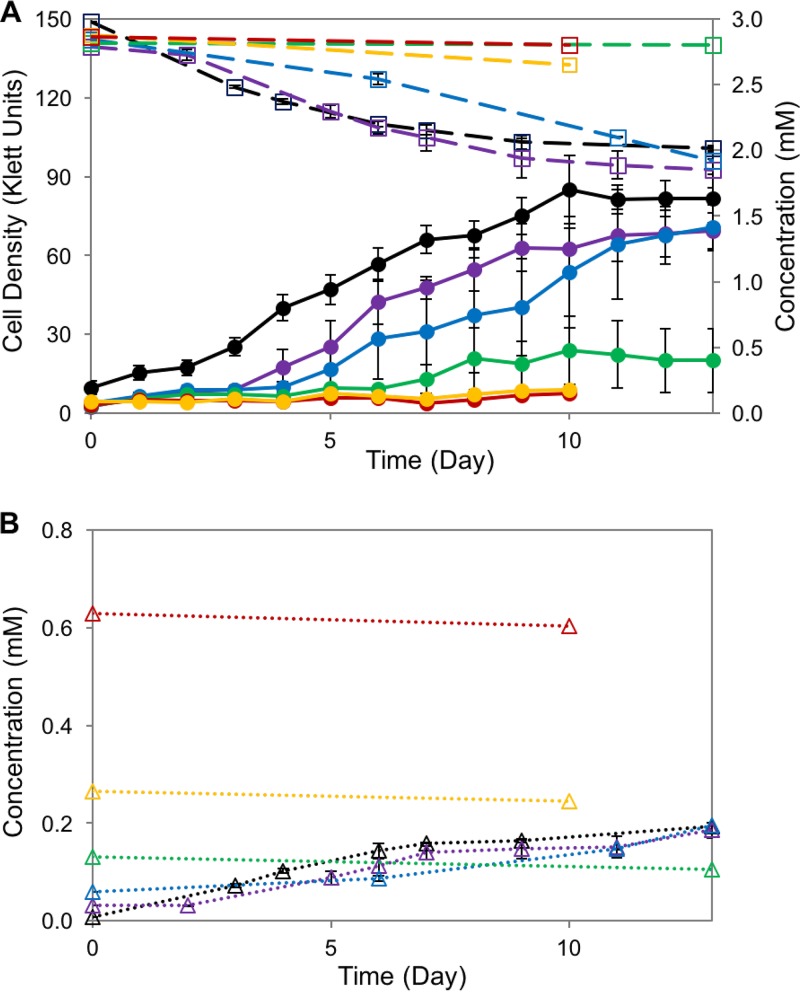
Effect of DMBQ on syringic acid degradation by SA008.1.07. The cultures received 3 mM syringic acid and various starting concentrations of DMBQ (black, 0 mM; violet, 0.03 mM; blue, 0.06 mM; green, 0.15 mM; yellow, 0.3 mM; red, 0.6 mM). (A) Solid lines show the cell density (in Klett units) (●); dashed lines show the syringic acid concentration (□). (B) DMBQ concentrations. As the initial concentration of DMBQ increased, cell growth and syringic acid degradation decreased. Cultures with DMBQ concentrations of 0.15 mM or greater showed no growth.

Since syringic acid was not totally degraded in the SA008.1.07 cultures (Fig. 3), we investigated whether the presence of DMBQ affected syringic acid metabolism by this strain. In one test of this hypothesis, we analyzed the photoheterotrophic growth of SA008.1.07 in cultures containing 3 mM syringic acid and various concentrations of DMBQ (Fig. 5). When the initial DMBQ concentration was 0.15 mM or above, we observed complete inhibition of growth (as scored by cell density) and of syringic acid degradation (Fig. 5). In experiments with initial DMBQ concentrations of less than 0.15 mM, growth and syringic acid degradation occurred, and extracellular DMBQ concentrations increased to about 0.19 mM. Thus, the results of this experiment suggested that, over the range of concentrations tested, DMBQ had an inhibitory effect on syringic acid degradation and cell growth. The inhibitory effect increased as the DMBQ concentration increased, suggesting that the buildup of DMBQ in medium containing syringic acid can prevent its total degradation by SA008.1.07. To test this hypothesis, we added 0.3 mM DMBQ (a concentration that approximates the amount found in stationary-phase syringic acid-grown cultures) to an SA008.1.07 culture when growth on syringic acid was detected (see Fig. S1 in the supplemental material). We found that the addition of 0.3 mM DMBQ arrested growth and blocked further syringic acid degradation in this culture compared to the findings for a control not receiving any added DMBQ.

To test whether the negative impact of DMBQ on growth was seen in cells grown in the presence of other aromatic substrates, we tested its effects on photoheterotrophic cultures grown on equimolar amounts of benzoic acid and 4-HBA. In this case, we found that addition of 0.3 mM DMBQ to growing SA008.1.07 cultures reduced the rates of growth and of aromatic degradation compared to those for a control not receiving DMBQ (Fig. S2). However, the extracellular DMBQ concentrations decreased in these cultures, suggesting a low rate of DMBQ degradation that was not evident in experiments with syringic acid. To test the effect of DMBQ on cells growing on a nonaromatic substrate, SA008.1.07 was grown on succinate with various concentrations of DMBQ (Fig. S3). In this case, a lag phase was observed when DMBQ concentrations were 0.06 and 0.3 mM, and complete growth inhibition was observed at 0.6 mM. There was also the apparent degradation of DMBQ in these cultures (Fig. S3). These results indicate that the inhibitory effect of DMBQ on growth or substrate utilization is not specific to cells that are using syringic acid as a sole organic carbon source. However, the inhibitory effect of exogenous DMBQ was more pronounced in cultures growing on aromatic substrates than in those growing on succinate as an organic carbon source. Furthermore, the evidence obtained with these experiments is not sufficient to determine whether DMBQ is in the syringic acid degradation pathway. For instance, a benzoquinone has been described to be a toxic intermediate in the degradation pathway of pentachlorophenol by Sphingobium chlorophenolicum (24). The decrease in the DMBQ concentration observed in experiments with 4-HBA and succinate could be a result of DMBQ either being slowly degraded or reacting with cellular components, as described for tetrachlorobenzoquinone in S. chlorophenolicum (24).

### Syringic acid degradation by R. palustris SA008.1.07 does not require the BAD pathway.

**FIG 6:**
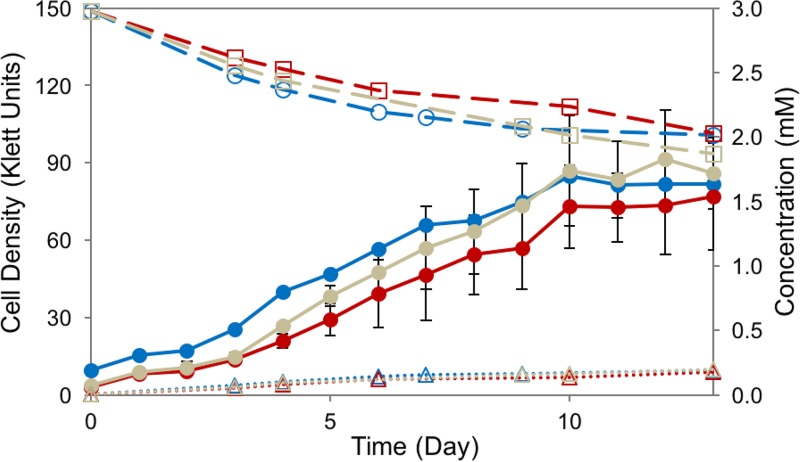
Photoheterotrophic degradation of syringic acid by R. palustris strains SA008.1.07 (blue), SAΔbadE (red), and SAΔhbaB (gray). Solid lines show the cell density (in Klett units) (●), dashed lines show the concentrations of syringic acid (□), and dotted lines show the DMBQ concentrations (Δ).

To date, the only known route for photoheterotrophic degradation of aromatic compounds in R. palustris is through the BAD pathway (19) (Fig. S4). To examine the role of the BAD pathway in syringic acid degradation by SA008.1.07, we created SAΔbadE, a mutant of this adapted strain lacking the benzoyl-CoA reductase gene. This deletion is sufficient to block the anaerobic degradation of all tested aromatic substrates in wild-type strain R. palustris CGA009 (19). We found that the SAΔbadE mutant strain lacks the ability to consume benzoic acid or 4-HBA, as expected (Table 1). However, we also found that SAΔbadE grows on syringic acid, exhibiting a behavior similar to that of the parent strain, SA008.1.07 (Fig. 6). We also examined the role of the peripheral HBA pathway, responsible for the conversion of 4-HBA into benzoyl-CoA (Fig. S4), in the growth of strain SA008.1.07 on syringic acid. To do this, we created SAΔhbaB, a mutant of SA008.1.07 which lacks the 4-hydroxybenzoyl-CoA reductase gene, which is known to be required for 4-HBA metabolism in R. palustris CGA009 (25). As expected, we found that the SAΔhbaB mutant lacks the ability to degrade 4-HBA, yet it can degrade benzoic acid (Table 1). As with the SAΔbadE mutant, we found that SAΔhbaB maintained the ability to grow on and degrade syringic acid (Fig. 6).

**TABLE 1:** Endpoint analysis of R. palustris SA008.1.07 *bad* and *hba* mutants grown in PM medium containing benzoic acid and 4-HBA^*a*^

Culture	Benzoic acid concn (mM)	4-HBA concn (mM)	Final cell density (Klett units)
SA008.1.07	ND	ND	172
SAΔbadE	1.36	1.45	18
SAΔhbaB	ND	1.43	81

a The PM medium contained benzoic acid at 1.41 mM and 4-HBA at 1.63 mM. ND, not detected.

From these experiments, we conclude that neither the peripheral HBA pathway nor the BAD pathway is required for the degradation of syringic acid by R. palustris SA008.1.07. This was a surprising result because the BAD pathway is the only known route for anaerobic aromatic metabolism in R. palustris (16, 19).

### Growth on syringic acid does not induce expression of BAD pathways in R. palustris SA008.1.07.

We used RNA sequencing (RNA-seq) to compare the global changes in transcript levels in cultures of SA008.1.07 anaerobically grown on syringic acid, 4-HBA, and succinate (Tables 2 and 3). Comparing growth on 4-HBA to growth on succinate revealed the expected increase in the transcript abundance of genes involved in the BAD pathway and the peripheral HBA pathway (Table 2). This is consistent with the above-mentioned finding that SA008.1.07 uses the BAD pathway for 4-HBA metabolism (18). However, the abundance of transcripts from these genes was much lower and mostly not significantly differentially expressed (*P > *0.05) when the growth of SA008.1.07 on syringic acid and succinate was compared (Table 2). Therefore, in addition to SA008.1.07 not needing the BAD and HBA pathways for growth on syringic acid (Fig. 6), the transcriptomics data show that growth in the presence of syringic acid does not induce the expression of known genes within the BAD and HBA pathways.

**TABLE 2:** Fold change in transcript abundance for genes predicted to be associated with the BAD and peripheral pathways when strain SA008.1.07 is anaerobically grown on 4-HBA or syringic acid compared to that when it is grown on succinate

Gene^*a*^	Name	Predicted product	Log_2_ fold change^*b*^	
			4-HBA to succinate	SA to succinate
*rpa0669*	*hbaA*	4-Hydroxybenzoate-CoA ligase	9.98*	3.63
*rpa0670*	*hbaB*	4-Hydroxybenzoyl-CoA reductase subunit	8.40*	3.01
*rpa0671*	*hbaC*	4-Hydroxybenzoyl-CoA reductase subunit	8.37*	2.58
*rpa0653*	*badI*	2-Ketocyclohexanecarboxyl-CoA hydrolase	7.84*	2.94*
*rpa0658*	*badE*	Benzoyl-CoA reductase subunit	7.68*	0.36*
*rpa0659*	*badF*	Benzoyl-CoA reductase subunit	7.39*	1.13
*rpa0660*	*badG*	Benzoyl-CoA reductase subunit	7.10*	0.95
*rpa0656*	*badC*	Alcohol dehydrogenase	6.82*	0.83
*rpa0654*	*badH*	2-Hydroxycyclohexanecarboxyl-CoA dehydrogenase	6.70*	2.00
*rpa0651*	*aliA*	Cyclohexanecarboxylate-CoA ligase	6.38*	1.00
*rpa0672*	*hbaD*	4-Hydroxybenzoyl-CoA reductase subunit	6.35*	0.83
*rpa0657*	*badD*	Benzoyl-CoA reductase subunit	6.09*	−0.40
*rpa0655*	*badR*	Benzoate anaerobic degradation transcription regulator	5.82*	1.37
*rpa0652*	*aliB*	Cyclohexanecarboxyl-CoA dehydrogenase	5.70*	0.87
*rpa0650*	*badK*	Cyclohex-1-ene-1-carboxyl-CoA hydratase	5.62*	0.69
*rpa0667*	*hbaF*	Inner membrane translocator	5.50*	0.98
*rpa0662*	*badB*	Ferredoxin	5.01*	0.55
*rpa0661*	*badA*	Benzoate-CoA ligase	4.91*	0.77
*rpa0668*	*hbaE*	ABC transporter subunit substrate-binding component	4.83*	0.95
*rpa0665*	*hbaH*	ABC transporter ATP-binding protein	4.67*	0.50
*rpa0673*	*hbaR*	Hydroxybenzoate anaerobic degradation regulatory protein	4.19*	0.46*
*rpa0666*	*hbaG*	ABC transporter ATP-binding protein	4.10*	−0.01
*rpa0664*	*badL*	Acetyltransferase	3.64*	0.04
*rpa3714*	*pimC*	Pimeloyl-CoA dehydrogenase large subunit	3.60*	0.67
*rpa3713*	*pimD*	Pimeloyl-CoA dehydrogenase small subunit	3.43*	0.23
*rpa0663*	*badM*	Transcriptional regulator BadM	3.02*	−0.03
*rpa3717*	*pimF*	Enoyl-CoA hydratase	2.63*	0.45
*rpa3715*	*pimB*	Acetyl-CoA acetyltransferase	2.58*	−0.22
*rpa3716*	*pimA*	AMP-dependent synthetase/ligase	2.53*	0.53

a Genes are sorted, in descending order, with respect to the log_2_ fold change in abundance when growth is on 4-HBA compared to that when growth is on succinate.

b SA, syringic acid. *, statistically significant difference (*P *< 0.05).

**TABLE 3:** Transcripts with the highest increase in abundance when strain SA008.1.07 is anaerobically grown on syringic acid compared to that when it is grown on succinate

Gene^*a*^	Name	Predicted product	Log_2_ fold change^*b*^	
			SA to succinate	4-HBA to succinate
*rpa0910*		Pirin family protein	9.70	1.95
*rpa2160*		3-Oxoacyl-ACP reductase	7.88*	−1.36
*rpa0909*	*wrbA*	NAD(P)H dehydrogenase (quinone)	6.80*	0.28
*rpa3619*	*vanA*	Aromatic ring-hydroxylating dioxygenase subunit alpha	6.66*	0.73
*rpa2717*		Hypothetical protein	6.27*	4.22
*rpa3621*	*vanB*	Oxidoreductase	6.11*	−0.29
*rpa0005*	*hppD*	4-Hydroxyphenylpyruvate dioxygenase	6.07	1.72*
*rpa3620*	*vanR*	GntR family transcriptional regulator	6.01*	−1.34
*rpa4284*		Polyisoprenoid-binding protein	5.95*	−0.71
*rpa4222*		Hypothetical protein	5.91*	1.32*
*rpa0319*		Hypothetical protein	5.90	7.58*
*rpa3329*		Hypothetical protein	5.87	3.42
*rpa1475*		Hypothetical protein	5.56	0.59
*rpa3631*		3-Oxoacyl-ACP reductase	5.52*	−1.20
*rpa4285*		Malonic semialdehyde reductase	5.51*	−1.73
*rpa3565*		l,d-Transpeptidase	5.39	−2.17*
*rpa3943*		Ferritin-like domain-containing protein	5.09*	0.55*
*rpa4394*		Isocitrate lyase	5.05*	6.21*
*rpa3308*		Ferritin-like domain-containing protein	5.03	1.12
*rpa0320*		4-Coumaroyl-homoserine lactone synthase	5.00	6.71*
*rpa1089*		Hypothetical protein	4.99	1.32
*rpa0214*		Hypothetical protein	4.92*	0.66*
*rpa4220*		l,d-Transpeptidase	4.92	0.68
*rpa4286*		Dioxygenase	4.90*	−2.03
*rpa2895*		Hsp20/alpha crystallin family protein	4.87	0.92*

a Genes are sorted, in descending order, with respect to the log_2_ fold change in abundance when growth is on syringic acid compared to that when growth is on succinate. Shaded rows indicate that the gene was explored in this study.

b SA, syringic acid. *, statistically significant difference (*P *< 0.05).

### Identification of a gene cluster required for syringic acid degradation by R. palustris SA008.1.07.

The global gene expression analysis was also used to identify genes with increased transcript abundance when SA008.1.07 was grown on syringic acid compared to that when it was grown on either 4-HBA or succinate (Table 3). Among the transcripts showing the largest increase in abundance are those derived from genes within a putative *vanARB* (*rpa3619*, *rpa3620*, *rpa3621*) operon. The *vanARB* genes are annotated as coding for the VanAB proteins and a GntR-family transcriptional regulator (VanR), homologues of which are known or proposed to act as repressors of the *vanAB* genes (27–30). The VanAB proteins are known or predicted subunits of an enzyme (VanAB) with aromatic ring-hydroxylating activity (16, 27). Homologues of VanAB are known or predicted to catalyze the oxidation of vanillic acid to protocatechuic acid and formaldehyde in Bradyrhizobium diazoefficiens (B. japonicum) (31) and *Pseudomonas* sp. strain HR199 (32). In addition, a VanAB homologue in a *Streptomyces* strain has the reported ability to demethylate syringic acid as well as other aromatic compounds (33). A global gene expression analysis of R. palustris SA008.1.07 grown aerobically on vanillic acid (Fig. S5; Table S3) confirmed the predicted role of the *vanAB* genes in aerobic vanillic acid degradation, since there was an increased abundance of transcripts encoding these genes along with others in a predicted pathway, with protocatechuic acid and formaldehyde being intermediate metabolites of aerobic vanillic acid degradation (Fig. S5).

The increased transcript abundance of the *vanARB* genes when SA008.1.07 was grown anaerobically on syringic acid was unexpected, given that the RNA was isolated from cells grown under anaerobic photoheterotrophic conditions. As described in Materials and Methods, for the RNA-seq experiments, the cultures were continuously bubbled with N_2_ and CO_2_ to avoid air entering the cultures. For all other experiments, the culture tubes were completely filled with medium, leaving no headspace, and when samples were withdrawn from the cultures for chemical analyses, the resulting headspace was flushed with argon gas to prevent the introduction of air into the cultures. These are standard techniques that have been successfully employed to grow anaerobic bacterial cultures and isolate oxygen-sensitive proteins in their active form (34).

We also monitored the abundance of diagnostic transcripts as a reporter for the presence of oxygen in our photoheterotrophic cultures. Analysis of the transcript abundance of photoheterotrophically grown cultures shows that there was a relatively low abundance of those encoding HemF, an oxygen-dependent coproporphyrinogen oxidase (RPA1514), or subunits of the low-affinity enzymes in the aerobic respiratory chain, such as cytochrome *bd* (RPA1319, RPA4452, and RPA4793-RPA4794) or cytochrome *aa_3_* oxidases (RPA1453, RPA4183, and RPA0831 to RPA0836) (Table S4). In contrast, transcripts from the following genes were, on average, ∼32-fold more abundant in the photoheterotrophic cultures than in those mentioned above which are associated with growth in the presence of oxygen: genes encoding subunits of the high-affinity cytochrome *cbb_3_* oxidase (RPA0015 to RPA0019); genes encoding the oxygen-independent coproporphyrinogen oxidase HemN (RPA1666); those needed for anaerobic growth in the light (15, 35), including ones that encode pigment biosynthetic enzymes or pigment-binding proteins of the photosynthetic apparatus (RPA1505 to RPA1507, RPA1521 to RPA1548, RPA1667-RPA1668, RPA3568); plus other genes whose induction requires the global anaerobic regulator FixK (RPA1006-RPA1007, RPA1554) (36, 37) (*P* = 0.01, unpaired *t* test) (Table S4). This analysis provides independent experimental evidence that the photoheterotrophic cultures used as a source of RNA or for other experiments in this study were anaerobic.

Nevertheless, to further test whether oxygen influences the ability of SA008.1.07 to degrade syringic acid, we performed additional experiments. First, when we tested SA008.1.07 for aerobic growth on the methoxylated aromatics syringic acid and vanillic acid (Fig. S6), we found that this adapted strain could not grow on syringic acid aerobically but could grow aerobically on vanillic acid. We also performed growth experiments in which additional steps were taken to eliminate oxygen from the medium. In one experiment, we used 100-ml serum bottles with PM medium (22) containing syringic acid and sealed them with rubber septa and aluminum crimp caps. We then flushed the PM medium with argon gas for 20 min and then applied vacuum to remove gases from the bottles and reflushed them with argon. This process was repeated three times to remove as much oxygen as possible. As a control that simulated the conditions used in the experiments described earlier, another group of 100-ml serum bottles was used, but in this case, the bottles were sealed without using the degassing procedure. SA008.1.07 was inoculated into both sets of bottles through sterilized syringes and needles. In these experiments, we observed no significant difference on the growth of SA008.1.07, the consumption of syringic acid, or the production of DMBQ between the degassed bottles and the nondegassed controls (Fig. S7), demonstrating that any traces of oxygen potentially present at the initiation of the incubations did not influence the ability of R. palustris SA008.1.07 to grow on syringic acid under anaerobic photoheterotrophic conditions. In a separate experiment, we used l-cysteine as a reducing agent and resazurin as an oxygen indicator (Fig. S8). Inoculation of R. palustris SA008.1.07 was performed after the resazurin was colorless, indicating the absence of oxygen. Syringic acid degradation and DMBQ production were observed, similar to the findings of the experiments performed with other techniques, further confirming that oxygen is not involved in the transformation of syringic acid.

**FIG 7:**
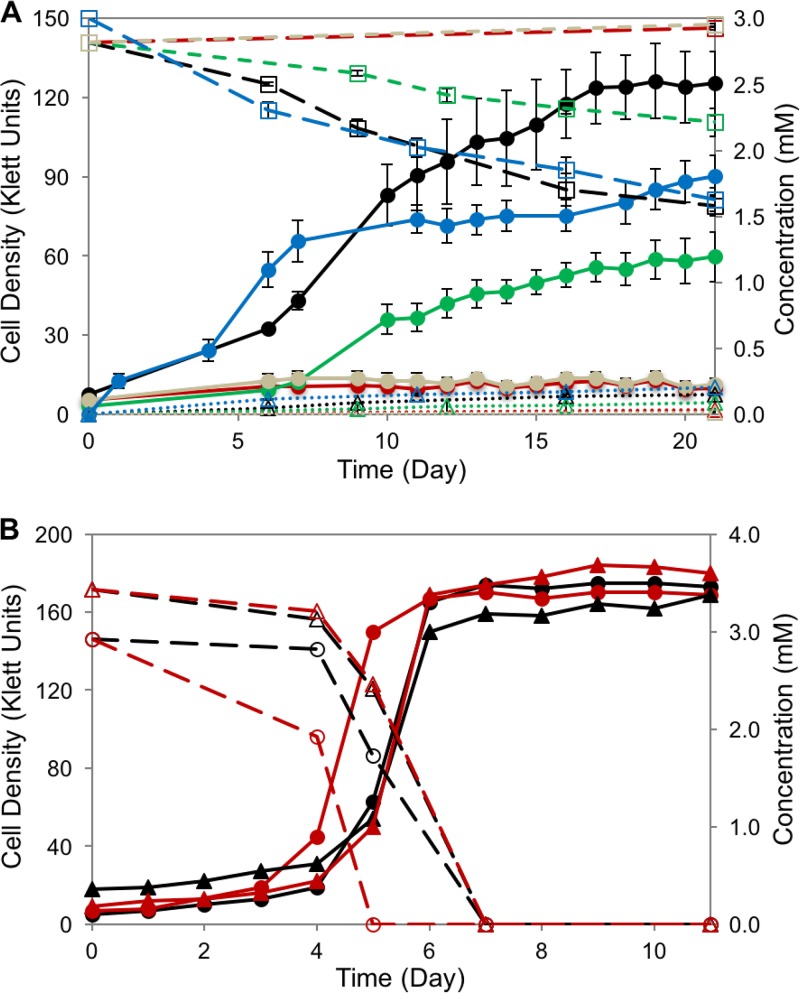
(A) R. palustris SAΔvan (red), a mutant culture of SA008.1.07 (black) with the *vanARB* operon deleted, does not grow on syringic acid. The complementation of *vanARB* on expression plasmids pBRVanARB and pBRVanAB restores syringic acid-degrading activity in SAΔvan/pBRvanARB (green) and SAΔvan/pBRvanAB (blue). The expression plasmid pBRVanARB does not impart syringic acid-degrading activity when inserted into wild-type strain CGA009 (A9/pBRvanARB; gray). Solid lines show growth (in Klett units) (●), dashed lines track the concentrations of syringic acid (□), and dotted lines track the DMBQ concentration (Δ). (B) Both R. palustris SA008.1.07 (black) and SAΔvan (red) grow on benzoic acid (circles) and 4-HBA (triangles). Solid lines show growth (in Klett units), and dashed lines indicate aromatic concentrations.

Based on these results, we proceeded to investigate whether the *vanARB* operon participated in anaerobic syringic acid degradation by SA008.1.07. To do this, we deleted the entire *vanARB* operon in SA008.1.07 (producing strain SAΔvan; Table 4) and found that this strain lost its ability to grow anaerobically on syringic acid (Fig. 7A). In addition, we found that transforming SAΔvan with a plasmid carrying either the wild-type *vanARB* operon or only wild-type *vanAB* (producing strain SAΔvan/pBRvanARB or SAΔvan/pBRvanAB, respectively; Table 4) under the control of a constitutive promoter rescued the ability of SAΔvan to grow on and degrade syringic acid under anaerobic conditions, although cell densities were lower than those for SA008.1.07 (Fig. 7A). Thus, we conclude that the *vanAB* genes in the R. palustris *van* cluster are required for the anaerobic degradation of syringic acid by SA008.1.07. In control experiments, we found that, as expected, the activities of the BAD and HBA aromatic pathways were not affected by the loss of *vanARB*, as SAΔvan was able to grow photoheterotrophically on 4-HBA or benzoic acid (Fig. 7B). Placing the same *vanARB* plasmid in the wild-type CGA009 strain (A9/pBRvanARB; Table 4) did not confer on this strain the ability to grow on syringic acid (Fig. 7A), indicating that yet to be identified genes outside this operon are required for syringic acid metabolism by SA008.1.07.

**TABLE 4:** Strains and plasmids used in this study

Strain or plasmid	Description	Source or reference
Strains		
* *E. coli		
DH5α	*supE44 lacU169* (ϕ80dΔ*lacZ*M15) *hsdR178 recA1 endA1 gyrA96 thi-1 relA1*	Invitrogen-THF
S17-1	C600::RP-4 2-(Tc::Mu) (Kn::Tn*7*) *thi pro hsdR* HsdM^+^ *recA*	55
NEB 5α	*fhuA2* Δ(*argF-lacZ*)*U169 phoA glnV44* ϕ80Δ(*lacZ*)M15 *gyrA96 recA1 relA1 endA1 thi-1 hsdR17*	NEB
* *R. palustris		
CGA009	Wild-type strain	22
SA008.1.07	Derivative of CGA009 able to grow on syringic acid	This work
SAΔbadE	Deletion of 3′ end of *badD*, whole *badE* gene, and 5' end of *badF* in SA008.1.07; ΩKn^r^ cassette insertion in place of deleted nucleotides	This work
SAΔhbaB	Deletion of *hbaB* in SA008.1.07	This work
SAΔvan	Deletion of *vanARB* operon in SA008.1.07	This work
A9/pBRvanARB	Gm^r^; CGA009 carrying pBRvanARB vector	This work
SAΔvan/pBRvanARB	Gm^r^; SAΔvan carrying pBRvanARB vector	This work
SAΔvan/pBRvanAB	Gm^r^; SAΔvan carrying pBRvanAB vector	This work
SAΔ2160	Deletion of *rpa2160* in SA008.1.07	This work
SAΔ4286	Deletion of *rpa4286* in SA008.1.07	This work
SAΔ1972	Deletion of *rpa1972* in SA008.1.07	This work
A9Δ1972	Deletion of *rpa1972* in CGA009	This work
Plasmids		
pSUP202	Mobilizable suicide plasmid	55
pK18mobsacB	*oriV oriT mob sacB* Kn^r^	53
pS202badE	3.7-kb fragment containing *badE* and most of surrounding genes *badD* and *badF* cloned into HindIII/BamHI sites of pSUP202	This work
pS202ΔbadE	Deletion of 2.1-kb fragment containing *badE*, 3' end of *badD*, and 5′ end of *badF* and insertion of 2.3-kb ΩKn^r^ cassette in pS202badE	This work
pK18hbaB	Kn^r^; 2.1-kb fragment containing *hbaB* and 800-bp flanking regions cloned into XbaI/HindIII sites of pK18mobsacB	This work
pK18ΔhbaB	Kn^r^; deletion of *hbaB* in pK18hbaB	This work
pKΔvanARB	Kn^r^; ∼1.5 kb upstream and ∼1.5 kb downstream flanking regions of *vanARB* operon cloned into the XbaI/HindIII sites of pK18mobsacB	This work
pBBR1MCS-5	IncA/C Gm^r^; broad-host-range cloning vector	54
pBRvanARB	Gm^r^; *vanARB* operon cloned into pBBR1MCS-5 vector	This work
pBRvanAB	Gm^r^; *vanA* and *vanB* genes cloned into pBBR1MCS-5 vector	This work

In addition to the genes in the *vanARB* operon, we also tested the effect of deleting two other genes that showed an increased transcript abundance during growth on syringic acid. One gene encodes an oxidoreductase and had one of the highest increases in transcript abundance (*rpa2160*), and the other gene was annotated as encoding a dioxygenase and had a lower increase in transcript abundance (*rpa4286*) (Table 3). Experiments with deletion mutants of SA008.1.07 lacking these genes, SAΔ2160 and SAΔ4286, respectively (Table 4), showed that neither deletion affected photoheterotrophic growth on syringic acid (Fig. S9), indicating that these genes are not required for the breakdown of syringic acid by SA008.1.07.

### Identification of mutations in strains adapted to grow on syringic acid.

In an attempt to identify additional mutations that could confer on R. palustris SA008.1.07 the ability to grow photoheterotrophically on syringic acid, we resequenced strain SA008.1.07 along with 16 other R. palustris isolates that had acquired the same metabolic ability by performing the same enrichment and isolation experiments described above (Table S5). When the genome sequences of this panel of isolates were compared to the genome sequence of R. palustris CGA009 (Table S6), only 4 mutations were found in the majority of the strains (Table 5). One mutation was an indel upstream of *rpa0746*, a gene annotated as encoding a cytochrome *c*-type cytochrome of unknown function. A second mutation was a frameshift in *rpa1972*, a gene annotated as encoding a two-component sensor histidine kinase, for which no function is known. The other two mutations were nonsynonymous, causing amino acid changes in *rpa2457*, which encodes a hypothetical protein, and *rpa3268*, which encodes the β subunit of RNA polymerase. No mutations were detected in the *vanARB* operon in any of the syringic acid-metabolizing strains that were sequenced.

**TABLE 5:** Mutations identified in more than half of the 17 adapted R. palustris strains conferring the ability of syringic acid degradation compared with the genome of CGA009

Position	Reference	Alteration	Mutation type	Amino acid change	Gene	Name	Function	Occurrence of mutation
826195	C	A	Substitution		Upstream *rpa0746*		Cytochrome *c*-type cytochrome	17 of 17 strains
2221051	TC	T	Frameshift at GLU242	Premature stop codon at 2220962	*rpa1972*		Two-component sensor histidine kinase	17 of 17 strains
2795227	G	A	Nonsynonymous	Glycine → aspartic acid	*rpa2457*		Hypothetical protein	16 of 17 strains
3685350	T	G	Nonsynonymous	Threonine → proline	*rpa3268*	*rpoB*	RNA polymerase β subunit	11 of 17 strains

We were unsuccessful in our attempts to delete *rpa2457* and *rpa3268* from SA008.1.07 using the methods used in this study, which is not surprising, since both of these genes have been shown to be essential for the growth of R. palustris (15). We successfully deleted *rpa1972* in both CGA009 and SA008.1.07, creating strains SAΔ1972 and A9Δ1972, respectively. To test the hypothesis that the observed frameshift in *rpa1972* altered the function of this predicted histidine kinase and somehow influenced syringic acid degradation by SA008.1.07, we evaluated the photoheterotrophic growth of both SAΔ1972 and A9Δ1972 on syringic acid. This experiment showed that deletion of *rpa1972* in CGA009 did not enable A9Δ1972 to grow on syringic acid, nor did deletion of this gene in SA008.1.07 prevent SAΔ1972 from growing on syringic acid (Fig. S10). Therefore, additional efforts are needed to identify single or synergistic combinations of mutations in SA008.1.07 or other adapted strains that contribute to anaerobic growth on syringic acid.

### Concluding remarks.

*meta*-Methoxylated aromatics are present at significant levels in the lignin of different plants and are potential sources of compounds for industrial applications. In this work, we isolated a strain of R. palustris that acquired the ability to use syringic acid as a growth substrate under photoheterotrophic conditions. Our strategy of incrementally exposing cultures to higher concentrations of syringic acid, while at the same time reducing the availability of the known growth substrates benzoic acid and 4-HBA, has been shown to be conducive to adaptation and the acquisition of new metabolic activities in R. palustris (38, 39) and other bacteria (40). Our analysis of this adapted strain, SA008.1.07, has provided important new knowledge on the bacterial metabolism of syringic acid. First, we found that syringic acid degradation does not occur through or induce the expression of the genes in the well-characterized BAD pathway. This finding makes syringic acid an aromatic compound whose photoheterotrophic metabolism does not utilize the BAD pathway in R. palustris. In addition, the increased abundance of *vanARB* transcripts in SA008.1.07 cultures grown in the presence of syringic acid and the requirement of *vanAB* for the growth of this adapted strain on this methylated aromatic provide evidence for a heretofore unknown role of the VanAB enzyme in the anaerobic metabolism of this compound. Since the previously reported function of *vanAB* is in the aerobic demethylation of vanillic acid (31, 32), our observations suggest that the VanAB enzyme may have an additional unrealized function under anaerobic conditions. Known homologues of VanAB are reported to contain an oxygen-sensitive iron sulfur cluster (32), so our findings reinforce the suggestion that additional experiments are needed to test the role of this enzyme in the anaerobic metabolism of syringic acid.

Our analysis of syringic acid metabolism by R. palustris SA008.1.07 sets the stage for further studies of the metabolism of this and other aromatics by this and other bacteria and for the evaluation of previously unexplored functions of the VanAB enzyme. Elucidating such novel pathways and metabolic functions could expand our ability to use microbial transformations of lignin and other renewable resources as biomass-based sources of compounds with potential uses in the energy, chemical, pharmaceutical, and other industries.

## MATERIALS AND METHODS

### Media.

All R. palustris strains were grown in PM medium (22), brought to pH 7 with sodium hydroxide, and sterilized by filtration. PM media with different organic carbon sources were prepared: PM-AcY contained 20 mM sodium acetate and 0.1% yeast extract, PM-succinate contained 10 mM succinic acid, and PM-aromatic was made with 3 to 3.5 mM aromatic compounds (unless otherwise indicated) and supplemented with 30 mM sodium bicarbonate. Escherichia coli strains were grown on LB medium (41). Molecular genetics-grade agar (Fisher Scientific, Fair Lawn, NJ) was added to the media at 1.5% to solidify the media, where noted. When necessary, the following reagents were used for cloning, selection, and propagation of modified strains: 10% (wt/vol) sucrose, 50 μg/ml kanamycin (Kn), 25 μg/ml ampicillin, and 20 μg/ml gentamicin. All chemicals for medium preparation were obtained from Fisher Scientific (Hampton, NH) or Sigma-Aldrich (St. Louis, MO) at purities suitable for molecular biology.

### Strains and plasmids.

The E. coli and R. palustris strains and plasmids used in this study are summarized in Table 4.

### Growth conditions.

To culture R. palustris, cells were streaked from glycerol freezer stocks onto PM-AcY agar plates and incubated aerobically at 30°C to obtain single colonies. A colony was transferred to 25 ml PM-AcY liquid medium and grown aerobically at 30°C. Aliquots (about 170 μl) from this aerobic culture were added to clear glass culture tubes (16 by 125 mm) containing PM-succinate. The tubes were completely filled to the brim with medium, sealed with a rubber septum, and incubated. Since the growing culture rapidly exhausts any oxygen available in the medium, this culturing technique has been demonstrated to efficiently create anaerobic culturing conditions in liquid medium (34). Photoheterotrophic growth was maintained at 30°C under illumination by incandescent tungsten lamps at ∼10 W/m^2^ and kept well mixed by a micro-magnetic stir bar (3 by 10 mm). These photoheterotrophic PM-succinate cultures were used as inocula for the photoheterotrophic experiments with the aromatic substrates, which were prepared by the procedure described above to generate anaerobic conditions in liquid medium. R. palustris growth in liquid cultures was monitored using a Klett-Summerson photoelectric colorimeter (Klett Manufacturing Co., New York, NY). Photoheterotrophic growth on solid medium was achieved by placing the plates in a sealed canister containing a GasPak EZ anaerobe container system (BD Biosciences, Franklin Lakes, NJ), which was placed under constant illumination and rotated daily.

### Analytical tests.

For chemical analysis, samples were taken periodically by aseptically piercing a rubber septum and withdrawing 200 μl of liquid culture. Following sampling, the headspace of the cultures was flushed with argon gas. Samples were passed through 0.22-μm-pore-size polyvinylidene difluoride membranes (Merck, KGaA, Darmstadt, Germany) to separate the cells from the medium, and the filtrates were frozen at −80°C until analysis.

Aromatic compounds were quantified by high-performance liquid chromatography (HPLC) using an LC-10ATvp solvent delivery module HPLC system (Shimadzu, Kyoto, Japan) with an SPD-M10Avp diode array detector (Shimadzu, Kyoto, Japan). Samples were prepared as described elsewhere (42). Aromatic compounds were separated using a C_18_ reversed-stationary-phase column and an isocratic aqueous mobile phase of methanol (30% [wt/vol]), acetonitrile (6% [wt/vol]), and 5 mM formic acid in water (64% [wt/vol]) at a flow rate of 0.8 ml min^−1^ (42). Aromatics and metabolic by-products were quantified using standard curves and UV absorbance. Standard curves were prepared using commercially purchased compounds (Sigma-Aldrich, St. Louis, MO) dissolved in dimethyl sulfoxide (DMSO).

Liquid chromatography-tandem mass spectrometry (LC-MS/MS) was used for identification of extracellular metabolic by-products, using a chromatography separation system similar to the one described above. Mass spectra were analyzed with a Thermo Q-Exactive mass spectrometer (Thermo Scientific, Waltham, MA). Standards were directly infused into the mass spectrometer. Spectra were acquired in the positive ionization mode with an MS/MS resolution of 17,500, an isolation width of 2.0 Da, and a normalized collision energy of 30%.

Nuclear magnetic resonance (NMR) was also used for identification of some metabolic by-products. For these tests, three consecutive 100-ml ethyl acetate (EtOAc) extractions were performed on 500 ml of spent medium at pH 6.5 to 7.0. The pH of the aqueous fraction was then lowered to ∼1 using 1 M hydrochloric acid. Additional organic compounds were extracted from this acidified aqueous fraction using three consecutive 100-ml dichloromethane (DCM) extractions. Both extractants were independently washed three times with saturated sodium bicarbonate (50 ml/extraction) and then twice with saturated sodium chloride (50 ml/extraction) and were then dried with sodium sulfate. Samples were filtered, and the solvent was evaporated. The NMR spectra of the extracted compounds were collected in acetone-*d*_6_ on a Bruker Avance 500-MHz spectrometer (Billerica, MA, USA) fitted with a cryogenically cooled 5-mm QCI (^1^H/^31^P/^13^C/^15^N) gradient probe with inverse geometry (proton coils were closest to the sample). The spectra were compared to those for high-purity standards from Sigma-Aldrich (St. Louis, MO).

Chemical oxygen demand (COD) was used to quantify soluble organic compounds and biomass (42), with measurements being performed on both filtered and unfiltered samples. The theoretical COD values for the various carbon sources used in this study are as follows: for benzoic acid, 240 mg of COD/mmol of substrate; for 4-hydroxybenzoic acid (4-HBA), 224 mg of COD/mmol of substrate; and for syringic acid, 288 mg of COD/mmol of substrate.

### Transcriptomic analysis (RNA-seq).

For transcriptomic analyses, R. palustris SA008.1.07 cultures were photoheterotrophically grown on PM–4-HBA, PM-syringic acid, or PM-succinate by bubbling with 95% N_2_ and 5% CO_2_ under constant illumination at 30°C to mid-log phase, when the RNA was harvested (43). For aerobic analyses, R. palustris SA008.1.07 was grown in shake flasks in the dark, using PM-vanillic acid. For each sample, rRNA was reduced (Ribo-Zero kit; Illumina), and a strand-specific library was prepared (TruSeq stranded total RNA sample preparation kit; Illumina). RNA from cultures grown anaerobically on PM–4-HBA and PM-syringic acid and aerobically on PM-vanillic acid and PM-succinate was processed and sequenced at the University of Wisconsin—Madison Biotechnology Center (Illumina HiSeq2500, 1 × 100 bp, single end). RNA from cultures grown anaerobically on PM-succinate was processed and sequenced at the U.S. Department of Energy (DOE) Joint Genome Institute (Illumina NextSeq, 2 × 151 bp, paired end). Three biological replicates were analyzed per growth condition. The paired-end FASTQ files were split into read 1 (R1) and read 2 (R2) files, and R1 files were retained for further analysis, as the other data contained only single-end reads. All FASTQ files were processed through the same pipeline. Reads were trimmed using the Trimmomatic (version 0.3) tool (44) with the default settings, except for a headcrop setting of 5, a leading setting of 3, a trailing setting of 3, a slidingwindow setting of 3:30, and a minlen setting of 36. After trimming, the reads were aligned to the R. palustris CGA009 genome sequence (GenBank assembly accession number NC_005296.1), using the Bowtie2 (version 2.2.2) program (45) with the default settings, except for the number of mismatches allowed, which was set to 1. Aligned reads were mapped to gene locations using the HTSeq (version 0.6.0) program (46) and the default settings, except that the reverse strandedness argument was used. The DESeq2 (version 1.22.2) program (47) was used to identify significantly differentially expressed genes from pairwise analyses, using a Benjamini and Hochberg (48) false discovery rate (FDR) of less than 0.05 as a significance threshold and/or a fold change of greater than 2. Raw sequencing reads were normalized using the number of reads per kilobase per million (RPKM) mapped reads. A full list of the gene transcripts normalized by the number of RPKM mapped reads is shown in Table S1 in the supplemental material. The accession number for the RNA-seq data in the Gene Expression Omnibus (GEO) database is GSE135630.

### Genome sequencing.

The genomic DNA of CGA009, SA008.1.07, and 16 other adapted strains which were capable of anaerobic degradation of syringic acid was isolated and purified (49). Genome sequencing was performed and the sequences were analyzed by the U.S. Department of Energy Joint Genome Institute on an Illumina NovaSeq (2 × 151 bp) sequencer. The resulting DNA reads were aligned to the R. palustris CGA009 genome (GenBank reference sequence NC_005296.1) using the short read alignment tool BWA (50). Single nucleotide polymorphisms and small indels were called using SAMtools mpileup and bcftools and then filtered using vcfutils.pl from the SAMtools package (51). The NCBI accession numbers for the sequences are PRJNA520130 to PRJNA520144, PRJNA537839, and PRJNA537840.

### DNA manipulation.

Purification of the PCR products was achieved using a QIAquick PCR purification kit (Qiagen, Hilden, Germany), and the PCR products were extracted and purified from the agarose gels using a Zymoclean gel DNA recovery kit (Zymo Research, Irvine, CA). A Zyppy plasmid miniprep kit (Zymo Research) was used to purify plasmid DNA. Sanger-based sequencing reactions, performed using a BigDye (v3.1) cycle sequencing kit (Applied Biosystems, Foster City, CA), were processed by the University of Wisconsin—Madison Biotechnology Center DNA Sequence Facility.

### Creation of mutants.

A fragment of DNA containing *badE* and ∼1.2 kb of flanking DNA up- and downstream of *badE* were PCR amplified, digested with HindIII and BamHI, and ligated into pSUP202 to create pS202badDEF. The *badE* coding region, 350 bp of the 3′ end of *badD*, and 350 bp of the 5′ end of *badF* were deleted from pS202badE by PCR with phosphorylated primers. The resulting PCR product was ligated to an ΩKn^r^ cassette (52) to create pS202ΔbadE. pS202ΔbadE was mobilized into R. palustris strains CGA009 and SA008.1.07 via conjugation with E. coli S17-1. Double crossovers were screened for Kn resistance and ampicillin sensitivity. The presence of the desired *bad* mutations was confirmed by sequencing the appropriate genomic region.

An in-frame, markerless deletion of *hbaB* was constructed using the suicide vector pK18mobsacB (53). Briefly, *hbaB* and ∼0.8 kb of flanking DNA up- and downstream of *hbaB* were PCR amplified from R. palustris genomic DNA, digested with XbaI and HindIII, and ligated into pK18mobsacB to generate pK18hbaB. The *hbaB* coding region was deleted from pK18hbaB by PCR with phosphorylated primers. The resulting PCR product was circularized by ligation to generate pK18ΔhbaB and transformed into E. coli DH5α. pK18ΔhbaB was introduced into R. palustris strains CGA009 and SA008.1.07 by electroporation. Double crossovers were screened for their ability to grow on PM-AcY with 10% sucrose and Kn sensitivity. The presence of the desired *hbaB* mutation was confirmed by sequencing the appropriate genomic region.

An in-frame, markerless deletion of *vanARB* was constructed in SA008.1.07 using the suicide vector pK18mobsacB (53). Briefly, ∼1.5 kb of the up- and downstream flanking regions of *vanARB* was PCR amplified from SA008.1.07 genomic DNA and assembled into pK18mobsacB using the NEBuilder HiFi DNA assembly master mix (New England Biolabs [NEB], Ipswich, MA) to create pKΔvanARB. The generation and confirmation of the *vanARB* mutant (SAΔvan) were performed using pKΔvanARB as described above. To generate plasmid pBRvan, a DNA fragment containing the *vanARB* operon was PCR amplified from SA008.1.07 genomic DNA, assembled into the pBBR1MCS-5 vector (54) using the NEBuilder HiFi DNA assembly master mix, and transformed into E. coli NEB 5α. After the construction of plasmid pBRvan was confirmed by DNA sequencing, it was introduced into R. palustris strains SA008.1.07 and SAΔvan by electroporation. In the same manner, plasmid pBRvanAB was constructed by assembly of the pBBR1MCS-5 vector with the *vanA* and *vanB* genes amplified from SA008.1.07 genomic DNA, confirmed, and transformed into SAΔvan. Transformants were selected on PM-AcY-gentamicin plates and confirmed by PCR and DNA sequencing. Gentamicin was added to maintain pBRvan and pBRvanAB.

Mutants with in-frame, markerless deletions of *rpa2160*, *rpa4286*, and *rpa1972* in SA008.1.07 and *rpa1972* in CGA009 were created in the same manner described above for SAΔvan, creating strains SAΔ2160, SAΔ4286, SAΔ1972, and A9Δ1972, respectively. The primers used for generating gene deletion mutants are shown in Table 6. A complete list of primers is shown in Table S2.

**TABLE 6:** Primers used in this study for construction of mutant strains

Function	Primer name	Sequence (5ʹ → 3ʹ)^*a*^	Target
*badE* deletion in SA008.1.07	badE-Up-Fw-HindIII	GATCTAAGCTTCACCGCCGCC	Upstream of *badE*
	badE-Dn-Rv-BamHI	GATCGGATCCGGATTGATGTTGATCGTC	Downstream of *badE*
	dbadFsmaI-f	CTTCCCCGGGCTAAGGGAGGAGG	pS202badDEF plasmid
	dbadDsmaI-r	TAGCCCCGGGACAGTTCGATCGACTTG	pS202badDEF plasmid
*hbaB* deletion in SA008.1.07	hbaB-XbaI-For	CCGTCTAGAACACCACGTCGTCTTCG	Upstream of *hbaB*
	hbaB-HindIII-rev	CACTAAGCTTACGGGCGAGCAGTG	Downstream of *hbaB*
	hbaB-ATW-for	TGGTCGACGGTATTGATAAGGTCACG	pK18hbaB plasmid
	hbaB-ATW-rev	CCATGACCAAAGTCAAGCAATCGTCAC	pK18hbaB plasmid
	Delta_hbaB_fwd	CAGCAGCAGCCCGACCTTCAA	SAΔhbaB
	Delta_hbaB_rev	GATCGCATTGGCGACGGCATTC	SAΔhbaB
*vanARB* operon deletion in SA008.1.07	pK18msb_fwd	AAAGTGCGTCGGGTGATG	pK18mobsacB plasmid
	pK18msb_rev	TGTTTCCAGTCGGTAGATATTCCACAAAACAGC	pK18mobsacB plasmid
	USVan_fwd	atatctaccgactggaaacaGTCGCTGGCTGGCTGCTG	Upstream of *vanARB*
	USVan_rev	cgtcggcggcCGTGGCCTCCCTGTGCATTATTG	Upstream of *vanARB*
	DSVan_fwd	ggaggccacgGCCGCCGACGTTCTGGGAC	Downstream of *vanARB*
	DSVan_rev	agcatcacccgacgcactttGGCGAGCGGCAACGTCTG	Downstream of *vanARB*
	van-fwd	GATCGATTCCATTCTGGTCTGGCTTCTGGT	SAΔvan
	van_rev	GACGTGATCTATCGTGGCGAGAAGGGTAAG	SAΔvan
*rpa2160* deletion in SA008.1.07	US2160_fwd	gcagccgcgcGATCGTTCTGTCCCTGATCGGAG	Upstream of *rpa2160*
	US2160_rev	agcatcacccgacgcactttGACCGGAGCGGTCCGTTG	Upstream of *rpa2160*
	DS2160_fwd	atatctaccgactggaaacaGTCGCCGGGGCAGGCGCC	Downstream of *rpa2160*
	DS2160_rev	cagaacgatcGCGCGGCTGCGCTCAAAGCTCC	Downstream of *rpa2160*
	RPA2160amp_fwd	TCAAACGCGGGGAGGTCAAG	SAΔ2160
	RPA2160amp_rev	ATTTCGACAAAGCTTCGCCGTTC	SAΔ2160
*rpa4286* deletion in SA008.1.07	US4286_fwd	atatctaccgactggaaacaGACGCCAAGGCGCATCCG	Upstream of *rpa4286*
	US4286_rev	actcggtgcgCGTCGCAATCTCCCTGATTTGAAATCG	Upstream of *rpa4286*
	DS4286_fwd	gattgcgacgCGCACCGAGTCATTCCGG	Downstream of *rpa4286*
	DS4286_rev	agcatcacccgacgcactttTCGTTCTCGAAGCTGTTGG	Downstream of *rpa4286*
	Delta4286_fwd	CACCTCGACCAGCTGTTCGTCCAG	SAΔ4286
	Delta4286_rev	CTTCGACACTATCTCGGTGGTGCACG	SAΔ4286
*rpa1972* deletion in SA008.1.07 or CGA009	RPA1972-US_fwd	taatgcagctggcacgacagGACCACACCACGGCTTCAG	Upstream of *rpa41972*
	RPA1972-US_rev	agtcccgatcCCAACACAAATCCTGCGC	Upstream of *rpa1972*
	RPA1972-DS_fwd	tttgtgttggGATCGGGACTTGCGGGAG	Downstream of *rpa1972*
	RPA1972-DS_rev	gactggctttctacgtgttcGCGGTCGAGGAAGCTCATG	Downstream of *rpa1972*

a Lowercase nucleotides represent sequences overlapping with either the pK18mobsacB plasmid or an upstream/downstream fragment of R. palustris.

### Data availability.

The accession number for the RNA-seq data in the Gene Expression Omnibus (GEO) database is GSE135630. The NCBI accession numbers for sequences are PRJNA520130 to PRJNA520144, PRJNA537839, and PRJNA537840.

## Supplementary Material

Supplemental file 1

Supplemental file 2
