# Supplementary material for: Anaerobic Degradation of Syringic Acid by an Adapted Strain of Rhodopseudomonas palustris
Source: Appl Environ Microbiol. 2020 Jan 21;86(3):e01888-19. doi: 10.1128/AEM.01888-19 (PMC6974649; doi:10.1128/AEM.01888-19)
Supplement: Supplemental file 1 [file zam003209574s1.pdf]

Supplemental Material for

Anaerobic degradation of syringic acid by an adapted strain of *Rhodopseudomonas palustris*

J. Zachary Oshlag,[1,2] Yanjun Ma,[1] Kaitlin Morse,[1] Brian T. Burger,[1] Rachelle A. Lemke,[1] Steven D. Karlen,[1,3] Kevin S. Myers,[1] Timothy J. Donohue,[1,4] and Daniel R. Noguera[1,2]

[1]Great Lakes Bioenergy Research Center, Wisconsin Energy Institute, University of Wisconsin, Madison WI 53726

[2]Department of Civil & Environmental Engineering, University of Wisconsin, Madison, WI 53706

[3]Department of Biochemistry, University of Wisconsin, Madison, WI 53706

[4]Department of Bacteriology, University of Wisconsin, Madison, WI 53706

Corresponding author: Daniel R. Noguera (dnoguera@wisc.edu)

SUPPLEMENTAL FIGURES

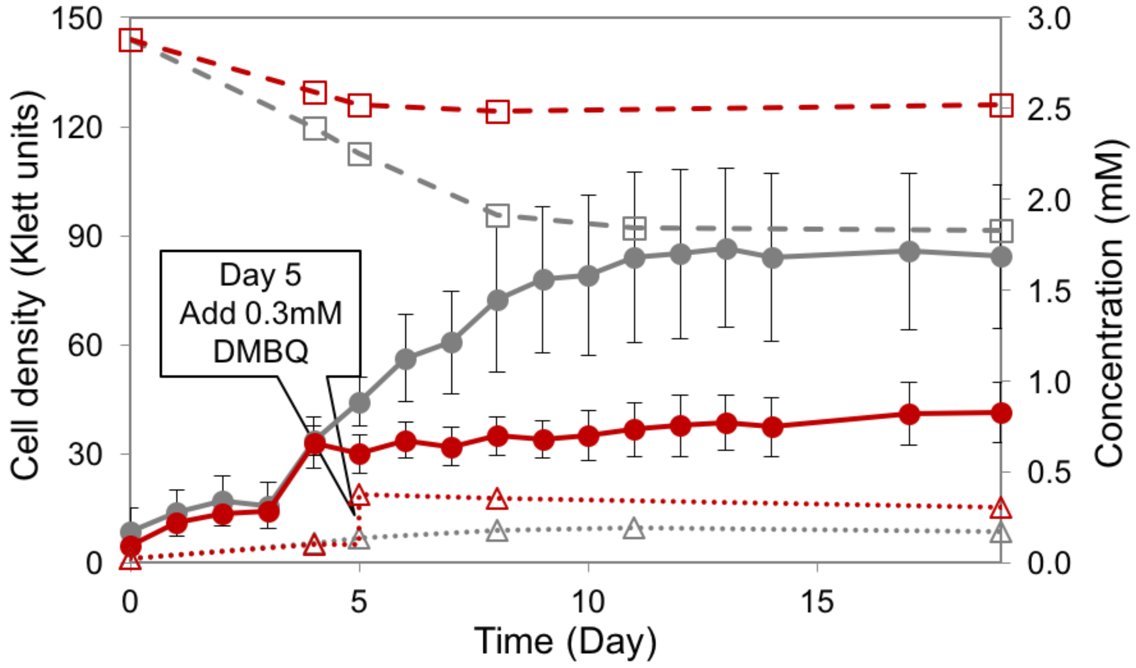

Figure S1. Effect of DMBQ addition on syringic acid degradation by *R. palustris* SA008.1.07. Solid lines show cell density (Klett units), dashes lines show syringic acid concentration, dotted lines show DMBQ concentration. Red lines indicate results for the DMBQ-containing culture and grey lines show results for a control culture not receiving DMBQ. DMBQ (0.3 mM) was added to the culture on Day 5. For this experiment, DMBQ was dissolved in DMSO, and the control culture was provided with DMSO only. Error bars represent standard deviation of experiments performed in triplicate.

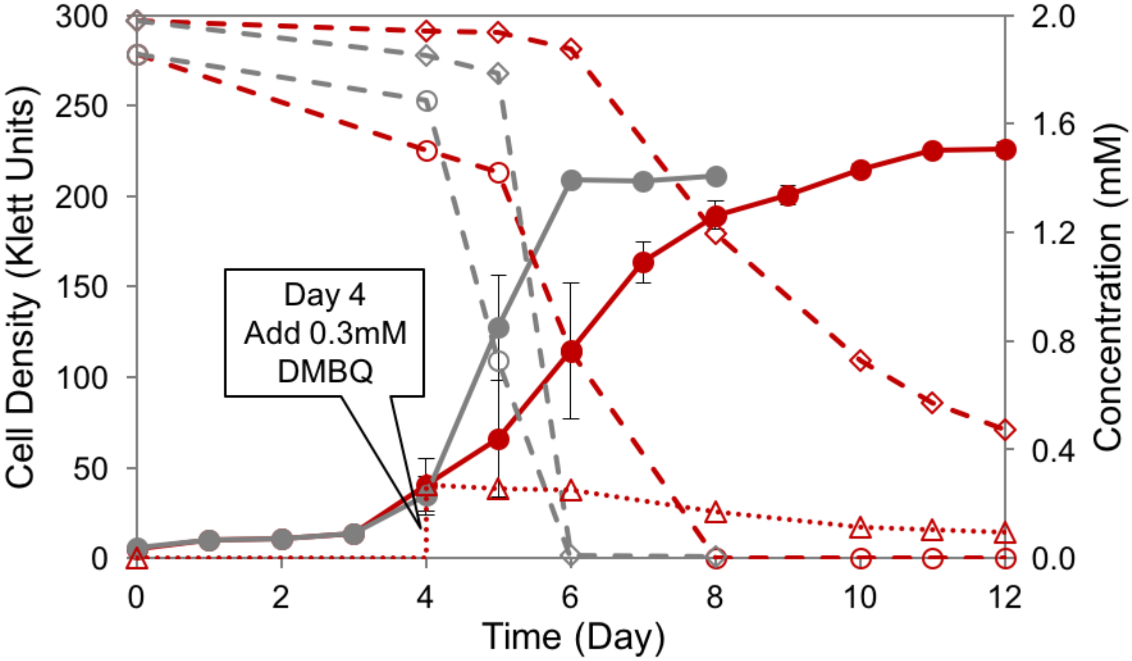

Figure S2. Effect of DMBQ addition on *R. palustris* SA008.1.07 growing on an equimolar amount of benzoic acid and 4-HBA (Initial concentration was 2 mM for each aromatic substrate). Solid lines are showing growth in Klett units (●), dashes tracking concentrations of benzoic acid (○), 4-HBA (◊), and dotted lines tracking DMBQ concentration (Δ). Red lines indicate results for the DMBQ-containing culture. DMBQ (0.3 mM) was added to the culture on Day 4. For this experiment, DMBQ was dissolved in DMSO. Parallel control cultures (in grey) received DMSO without DMBQ.

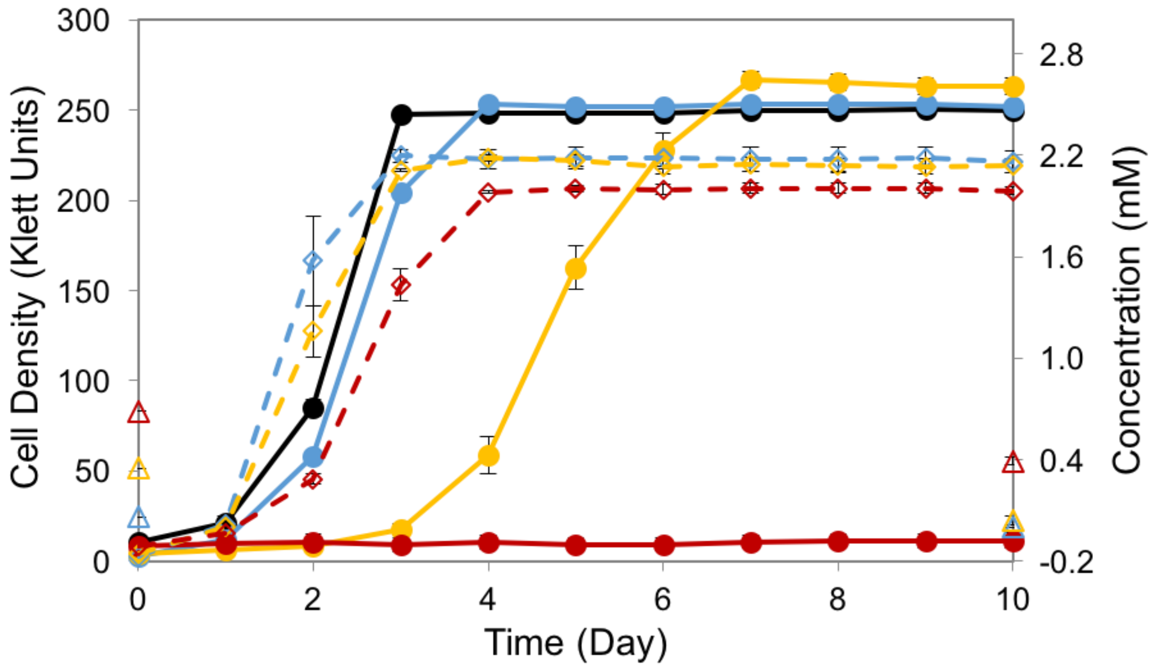

Figure S3. Effect of DMBQ on SA008.1.07 cultures grown on succinate. Solid lines show cell density of cultures received 10 mM succinate and various starting concentrations of DMBQ (black 0 mM, blue 0.06 mM, yellow 0.3 mM, red 0.6 mM). For these experiments, DMBQ was dissolved in DMSO. Dashed lines are showing growth of control cultures received corresponding amount of DMSO without DMBQ. Triangles (Δ) denote concentrations of DMBQ at the beginning and end of the experiment.

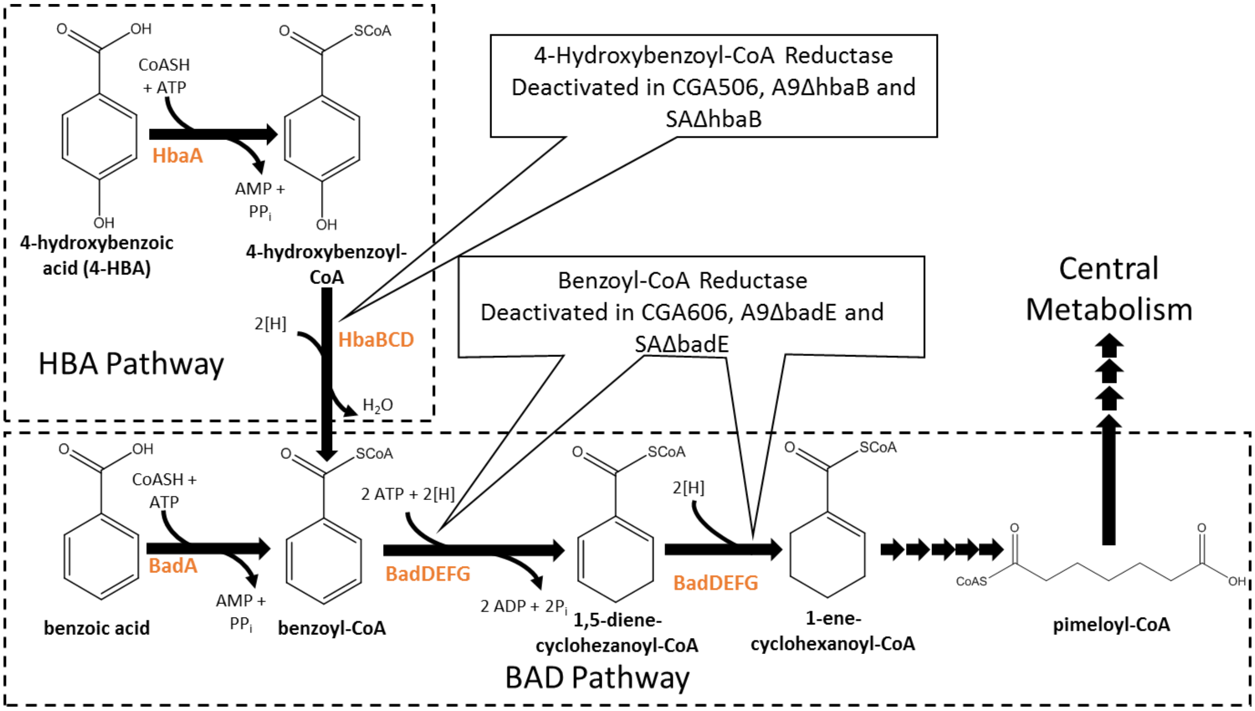

Figure S4. 4-Hydroxybenzoic acid (HBA) and benzoic acid degradation (BAD) pathways. These are the only previously established routes for anaerobic degradation of aromatic acids by *R. palustris.* HbaBCD and BadDEFG are oxygen sensitive enzymes.

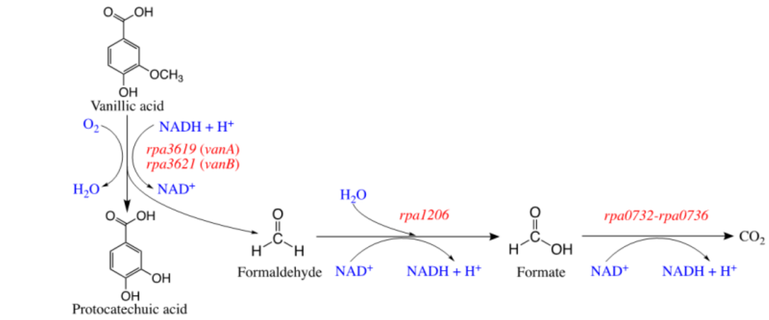

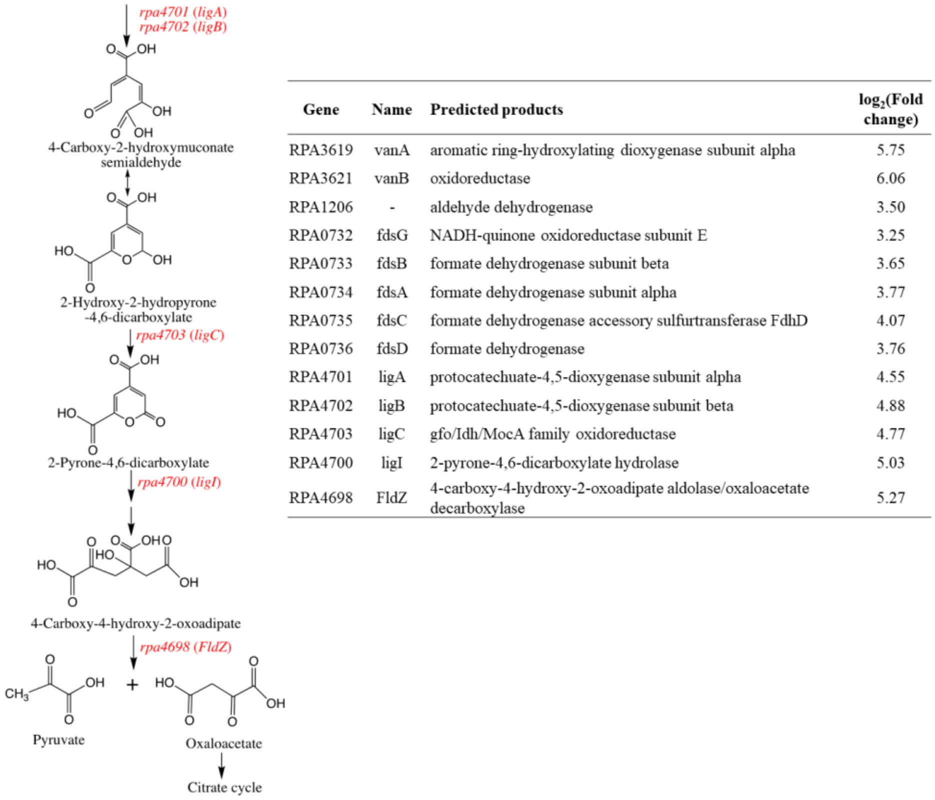

Gene	Name	Predicted products	$\log_2$(Fold change)
RPA3619	vanA	aromatic ring-hydroxylating dioxygenase subunit alpha	5.75
RPA3621	vanB	oxidoreductase	6.06
RPA1206	-	aldehyde dehydrogenase	3.50
RPA0732	fdsG	NADH-quinone oxidoreductase subunit E	3.25
RPA0733	fdsB	formate dehydrogenase subunit beta	3.65
RPA0734	fdsA	formate dehydrogenase subunit alpha	3.77
RPA0735	fdsC	formate dehydrogenase accessory sulfurtransferase FdhD	4.07
RPA0736	fdsD	formate dehydrogenase	3.76
RPA4701	ligA	protocatechuate-4,5-dioxygenase subunit alpha	4.55
RPA4702	ligB	protocatechuate-4,5-dioxygenase subunit beta	4.88
RPA4703	ligC	gfo/Idh/MocA family oxidoreductase	4.77
RPA4700	ligI	2-pyrone-4,6-dicarboxylate hydrolase	5.03
RPA4698	FldZ	4-carboxy-4-hydroxy-2-oxoadipate aldolase/oxaloacetate decarboxylase	5.27

Figure S5. Global gene expression analyses during aerobic degradation of vanillic acid by of *R. palustris SA008.1.07* results in overexpression of *vanAB* and other genes consistent with a hypothetical degradation pathway involving O-demethylation of vanillic acid (presumably by VanAB) with production of formaldehyde and protocatechuic acid acid. Log_2 (Fold change) represents the ratio of gene expression when growing on vanillic acid compared to succinic acid (See supplementary data set, Table S3).

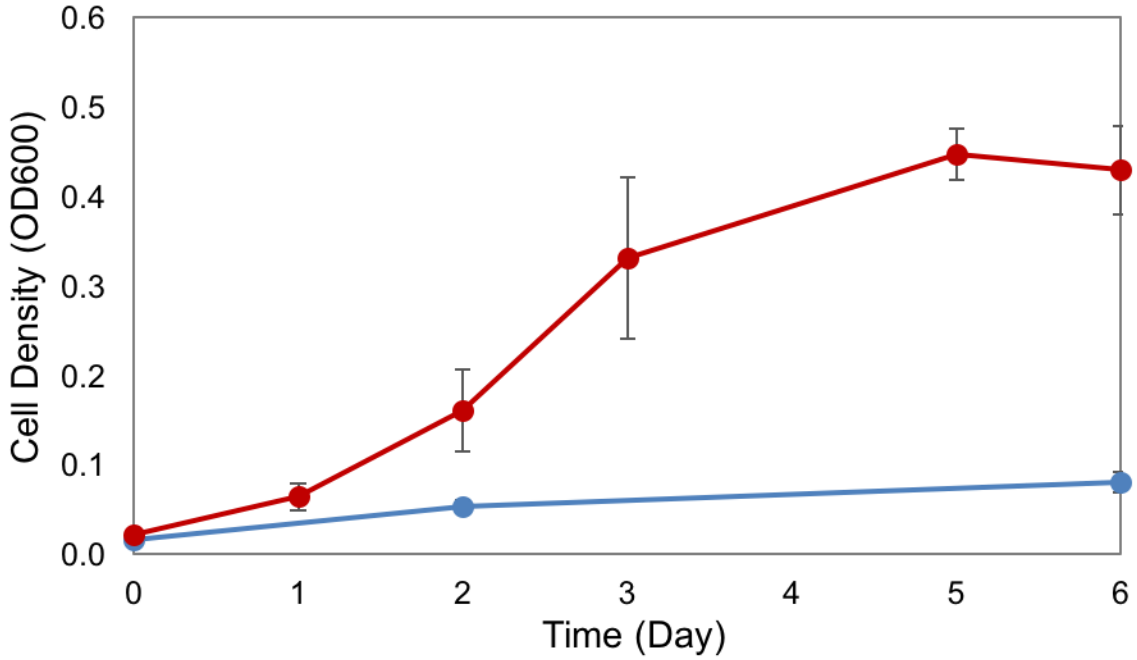

Figure S6. Aerobic growth of SA008.1.07 in 3 mM syringic acid (blue line) or vanillic acid (red line). At the end of experiment, syringic acid was not consumed, while vanillic acid was completely consumed.

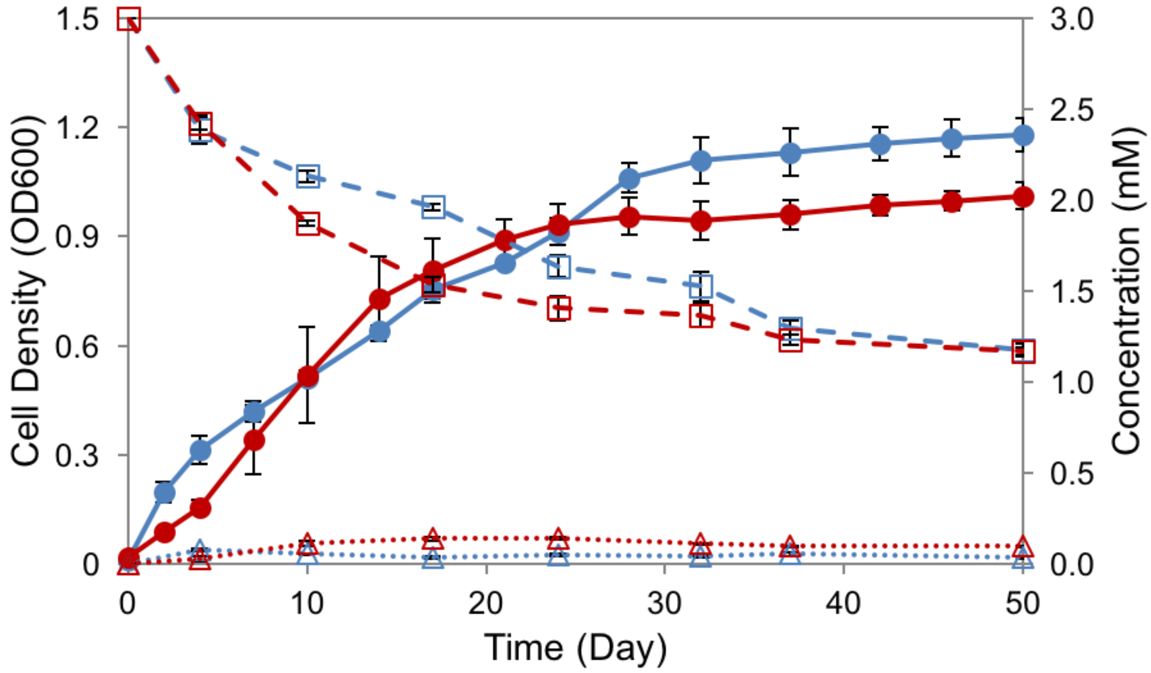

Figure S7. Cultures of SA008.1.07 in 3 mM syringic acid, grown on degassed (blue) and non-degassed (red) serum bottles. Solid lines are showing cell density in OD600 (●), dashes tracking concentrations of syringic acid (□), and dotted lines tracking DMBQ concentration (Δ).

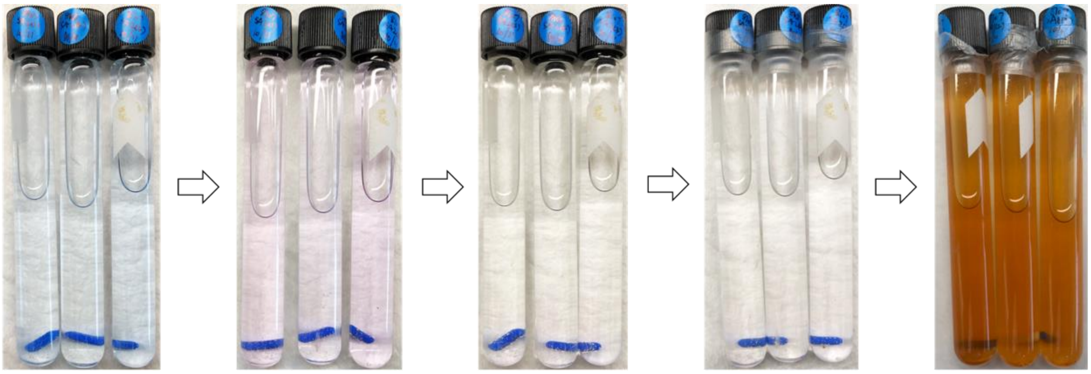

Figure S8. Anaerobic syringic acid degradation experiment using 1 mM L-cysteine as reducing agent and 1.5 μM resazurin as oxygen indicator. PM-syringic acid media (3 mM syringic acid), with L-cysteine and resazurin, was added to culture tubes and the tubes were sealed with rubber stoppers and screw caps. (A) The color of the media was initially light blue. (B) The media turned pink after about 30 min, indicating consumption of oxygen by L-cysteine. While the resazurin indicator was pink, the media was degassed by repeatedly applying vacuum and flushing the media with argon gas. (C) The resazurin indicator turned colorless after degassing and leaving the tubes on bench for about 3 hours; this indicated complete removal of oxygen from the media. (D) *R. palustris* SA008.1.07 was inoculated with a syringe, preventing any input of oxygen into the tubes. This was verified by the media remaining colorless after inoculation. (E) Cultures were incubated at 30 °C in front of the light. In this experimental setting, SA008.1.07 grew on syringic acid and the media acquired the typical color that is indicative of DMBQ accumulation. During this experiment, 1.3 mM of syringic acid was removed and 0.03 mM of DMBQ accumulated.

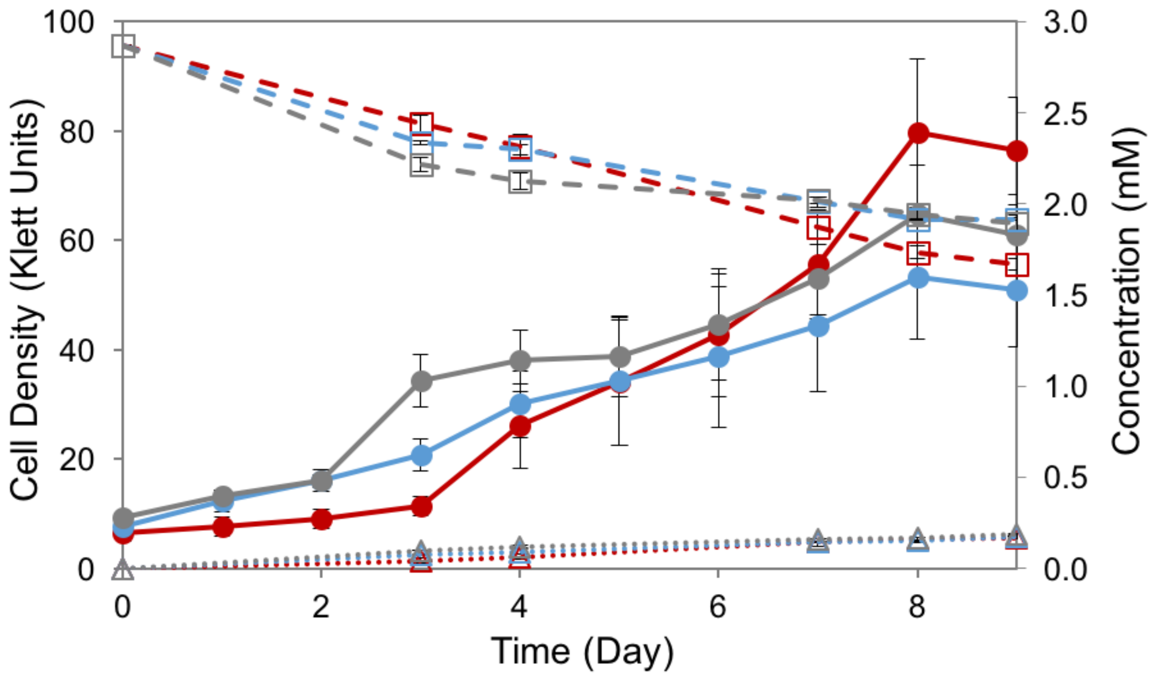

Figure S9. Cultures of *R. palustris* in 3 mM syringic acid. Growth and syringic acid consumption phenotype of SA008.1.07 (red) matches that of deletion strains SAΔ2160 (blue) and SAΔ4286 (grey). The genes that were deleted in these strains *rpa2160* and *rpa4286* do not appear to be necessary for growth of SA008.1.07 on syringic acid. Solid lines are showing growth in Klett units (●), dashes tracking concentrations of syringic acid (□), and dotted lines tracking DMBQ concentration (Δ).

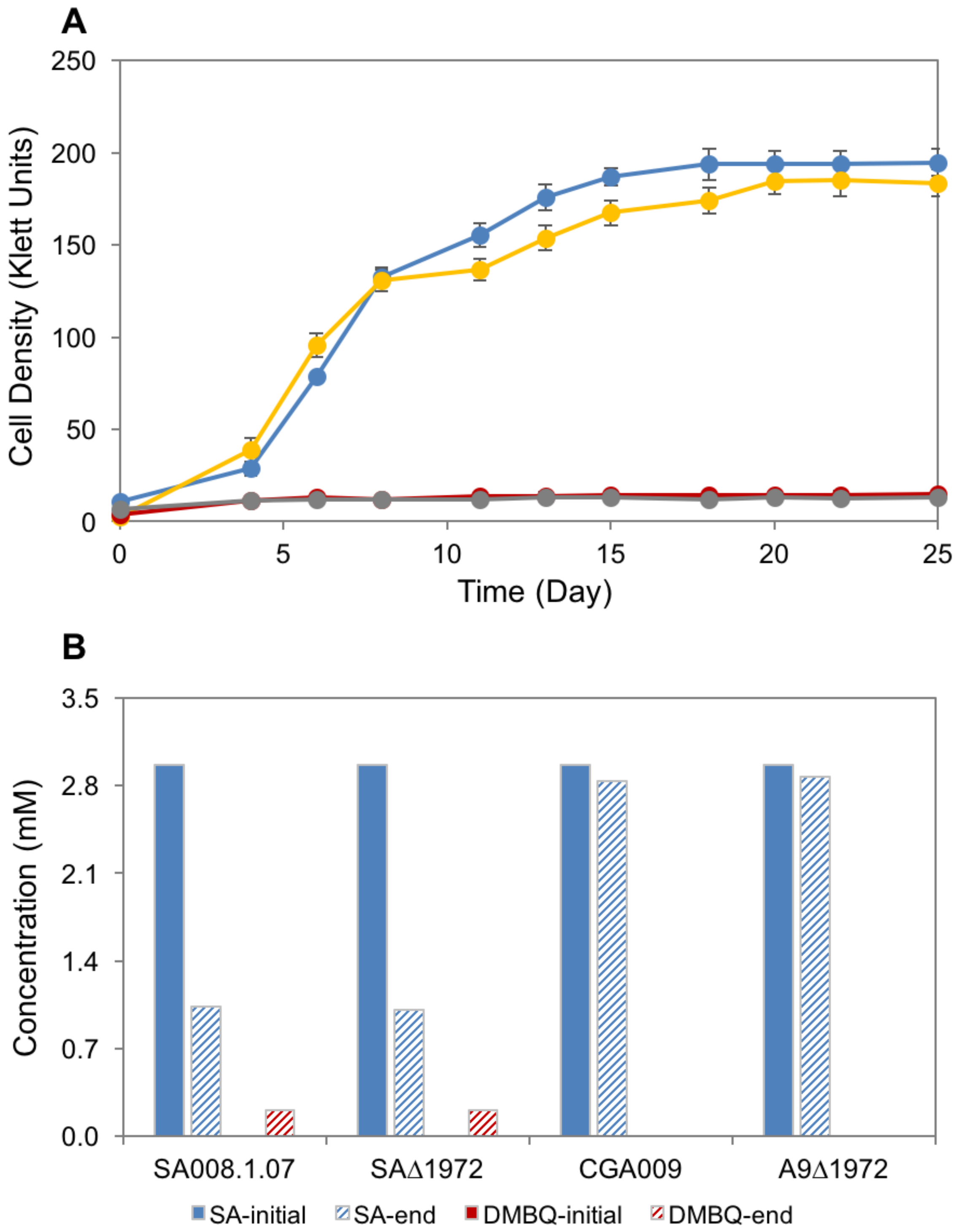

Figure S10. Cultures of *R. palustris* in 3 mM syringic acid. (A) Growth and syringic acid consumption phenotype of SA008.1.07 (blue) and CGA009 (red) matches that of deletion strains SAΔ1972 (yellow) and A9Δ1972 (grey), respectively. (B) Concentration of syringic acid (SA, blue bars) and DMBQ (red bars) in the initial- and end-points of the cultures.
